# Modelling and Design of Pre-Equalizers for a Fully Operational Visible Light Communication System

**DOI:** 10.3390/s23125584

**Published:** 2023-06-14

**Authors:** Murat Bostanoglu, Yaser Dalveren, Ferhat Ozgur Catak, Ali Kara

**Affiliations:** 1 Department of Electrical and Electronics Engineering, Gazi University, Ankara 06570, Turkey; murat.bostanoglu@gazi.edu.tr (M.B.); akara@gazi.edu.tr (A.K.); 2 Department of Electrical and Electronics Engineering, Atilim University, Incek Golbasi, Ankara 06830, Turkey; yaser.dalveren@atilim.edu.tr; 3 Electrical Engineering and Computer Science, University of Stavanger, 4021 Stavanger, Norway

**Keywords:** visible light communication, light fidelity, light emitting diode, optical wireless communication, digital pre-equalization

## Abstract

Nowadays, Visible Light Communication (VLC) has gained much attention due to the significant advancements in Light Emitting Diode (LED) technology. However, the bandwidth of LEDs is one of the important concerns that limits the transmission rates in a VLC system. In order to eliminate this limitation, various types of equalization methods are employed. Among these, using digital pre-equalizers can be a good choice because of their simple and reusable structure. Therefore, several digital pre-equalizer methods have been proposed for VLC systems in the literature. Yet, there is no study in the literature that examines the implementation of digital pre-equalizers in a realistic VLC system based on the IEEE 802.15.13 standard. Hence, the purpose of this study is to propose digital pre-equalizers for VLC systems based on the IEEE 802.15.13 standard. For this purpose, firstly, a realistic channel model is built by collecting the signal recordings from a real 802.15.13-compliant VLC system. Then, the channel model is integrated into a VLC system modeled in MATLAB. This is followed by the design of two different digital pre-equalizers. Next, simulations are conducted to evaluate their feasibility in terms of the system’s BER performance under bandwidth-efficient modulation schemes, such as 64-QAM and 256-QAM. Results show that, although the second pre-equalizer provides lower BERs, its design and implementation might be costly. Nevertheless, the first design can be selected as a low-cost alternative to be used in the VLC system.

## 1. Introduction

### 1.1. Preamble

Wireless communication is attracting more and more attention with the advancement of technology. With this increasing attention for wireless communication, available radio spectrum below 10 GHz has become insufficient [1]. Despite this limitation, Radio Frequency (RF)-based communication is still the most popular method used in wireless communication. However, Optical Wireless Communication (OWC) attracts increasing attention to overcome this limitation. OWC uses visible (VL), infrared (IR) and ultraviolet (UV) bands, which are in the upper parts of the electromagnetic spectrum and have features, such as being license-free and having high bandwidth [2].

Visible Light Communication (VLC) is an optical wireless communication method that uses visible light as a medium to transmit data. It should be noted that the visible light spectrum is a part of the electromagnetic spectrum, which is visible to the human eye and has a wavelength spectrum from 380 nm to 780 nm. Since the visible light source can be used for both illumination and communication, it provides extra power and cost savings compared to using two separate sources [3,4]. This feature makes the visible light spectrum more advantageous than other optical spectra, such as IR. Moreover, VLC has various advantages over RF-based communication, such as access to a wide unlicensed spectrum, as well as immunity to electromagnetic interference and security issues. These advantages make VLC quite attractive. Therefore, VLC has a wide range of uses, such as vehicular communication [5], defense and security industry [6], underwater communication [7], internet-of-things [8], and many others.

In VLC, there are different types of light sources, such as the Light-Emitted Diode (LED) and the Laser Diode (LD) [9]. However, because of their higher reliability, LEDs are mostly used in various applications based on data communications, and thus many other demonstrations of data transmission can also be performed [10]. Although VLC offers a wide bandwidth, the bandwidth of the LEDs used in the application is one of the key items to limit the reachable data rate. Although phosphor is widely used in lighting applications, the direct modulation speed of a LED with phosphor is usually limited to about 1 MHz [11]. This bandwidth limitation principally results from the slow temporal response of the phosphor [12]. In order to eliminate this limitation, equalization methods are employed.

Basically, there are two main types of equalization methods: (a) pre-equalizer at the transmitter side and (b) post-equalizer at the receiver side. Moreover, these methods can be applied in two different ways: digital or analog. On one hand, digital pre-equalizer methods have been developed through the different types of techniques, such as adding weights to subcarriers, or the use of finite impulse response (FIR) filters [13,14,15,16,17,18,19,20,21,22]. Besides, analog pre-equalizer methods have been developed using techniques, such as the cascaded constant-resonance amplitude equalizer and the multiple-resonant-driving equalization technique [23,24]. On the other hand, post-equalizer methods are also frequently used. Some analog post-equalizer methods have been presented using constant first order RC filter [25] or continuous time linear equalization (CTLE) technique with switchable response [26]. In addition, digital and analog post-equalizers are presented together by using the digital FIR filter and analog RC filter simultaneously [27].

Although various equalizer methods have been presented in the literature, some difficulties are expected to appear in their implementations. First, the implementation and flexibility of analog equalizer methods might be difficult and limited in terms of application methods [21]. Second, post-equalizers are disadvantageous in terms of power allocation, since the equalization is performed on the receiver side. Therefore, with post-equalizers, the ranges of Analog Digital Converter (ADC) and Digital Analog Converter (DAC) may not be used at full scale. In contrast, pre-equalization can efficiently extend LED bandwidth without sacrificing the received signal power since gain can be applied after equalization [20]. Thus, it can be deduced that digital pre-equalizers are the most suitable method to implement an equalizer in a VLC system due to their simple and reusable structure.

### 1.2. Related Work and Contributions

In the literature, several digital pre-equalizer methods have been proposed for VLC systems [13,14,15,16,17,18,19,20,21,22]. In [13], in order to improve the modulation speed of 1 MHz bandwidth white LED communication, digital FIR and matched filters were used. With the proposed approach, it was demonstrated that a data transmission rate of 20 Mbit/s can be achieved using four-level amplitude shift keying. In [14], a VLC system based on the use of digital adaptive equalizers was proposed. Experimental results demonstrated that the proposed system can offer more than 100 Mbit/s data transmission rates. In [15], to achieve higher transmission speeds, a power exponential software pre-equalization was applied to the VLC system based on a white LED. By means of experimental demonstrations, a data transmission rate of 2.08 Gbit/s over a 1 m distance was achieved with a bit error rate (BER) under 3.8 × 10^−3^. In [16], an automotive headlight VLC system with pre-equalization was proposed. Experimental results showed that a data transmission rate of 427.5 Mbit/s at a distance of 1.2 m can be achieved with BER under 3.8 × 10^−5^. In [17], a digital pre-equalization scheme that uses a luminous feedback signal at the transmitter side of a VLC system mode was proposed to mitigate the non-linearities and low-pass effect inherent to orthogonal frequency division multiplexing (OFDM)-based VLC systems. In [18], a simple approach to propose a digital pre-equalization was described. The performance of the approach was investigated by analyzing the number of time points required to represent each pre-equalized bit. In [19], a digital equalizer based on a scheme that consists of two square-root-raised-cosine filters, and a FIR filter was used to compensate the channel fading at high frequency induced by LEDs in VLC. It was shown that a transmission rate of 120 Mb/s can be realized when the 3-dB bandwidth of the LED is 13 MHz. In [20], a distributed digital pre-equalization for VLC systems based on OFDM was presented. It was demonstrated that a data transmission rate of 976.6 Mbit/s can be achieved by using distributed digital pre-equalization. In [21], a LED frequency response model was used to design a pre-equalizer. It was demonstrated that a data transmission rate of 180 Mbit/s over 1.5 m with a BER around 0.010 can be achieved using on-off keying modulation. In [22], an adaptive digital pre-equalization scheme based on a deep learning model was proposed for VLC channels. According to the experimental results, it was concluded that, with the proposed scheme, high data transmission rate can be achieved with a BER under 10 × 10^−6^.

As can be inferred from the relevant works reported in the literature, the performance of a digital pre-equalizer to be implemented in a VLC system is mostly examined in terms of BER and throughput performances in an Additive White Gaussian Noise (AWGN) channel. However, when the performance assessment of different digital pre-equalizers is concerned, it is necessary to take several factors into account. First of all, for a reliable assessment, each of the pre-equalizers needs to be tested with the same algorithm. Besides, it is necessary to use the same test environment while comparing the pre-equalizers. Furthermore, although LED is the most important component limiting the bandwidth in a VLC system, other components, such as ADC, DAC, and analogous front-ends of the VLC transmitter and VLC receiver, have significant effects on the channel. Therefore, in order to achieve more realistic results, the performance of a digital pre-equalizer implemented in a complex algorithm, that is compatible with a standard, by considering a channel containing the frequency responses of the components, can be investigated. Recently, the IEEE 802.15.13 standard has been approved for optical wireless communications applications, supporting data transmission rates of multiple Gbit/s [28]. Yet, there is no study in the literature that examines the implementation of pre-equalizers in a realistic VLC system based on the IEEE 802.15.13 standard.

In this study, it is aimed to propose digital pre-equalizers to be used in VLC systems based on the IEEE 802.15.13 standard. To this end, a fully operational VLC system was modeled in MATLAB in which the designed the pre-equalizers are utilized. That is, in order to model a realistic VLC channel for the system, a real 802.15.13-compliant VLC system was utilized in pre-equalization works. In this system, firstly, the signal recordings were collected. The recordings were then post-processed, and the channel response of the system was obtained by the channel estimation training sequence (CETS). Using the obtained channel response, a FIR filter was implemented to model the channel. Next, the channel model was added to the modelled VLC system. Furthermore, two different digital pre-equalizers were designed. In the first design, it was intended to utilize the inverse of the gain responses of the subcarriers obtained from the CETS. For this design, a post-process approach is applied in a software environment to remove CETS from the recordings. In the second design, on the other hand, a post-process is not applied, as it is based on the use of sinusoidal signals collected from a real setup. Both designs were then tested on the system. Their feasibility was comparatively assessed in terms of the BER performance under bandwidth-efficient modulation schemes, such as 64-QAM and 256-QAM. From the results, it was obtained that the system with the second pre-equalizer provides better BER performance. Yet, the first equalizer offers a low-cost option to be used in the VLC system.

The main contributions of this study can be summarized as follows:

- Two different digital pre-equalizers are proposed to be used in VLC systems based on the IEEE 802.15.13 standard.
- The effects of digital pre-equalizers on a VLC system compatible with the IEEE 802.15.13 standard are discussed for the first time in the literature.

The rest of the article is organized as follows: In Section 2, an overview of the VLC system modeled in MATLAB is presented. Here, the basic structures of the transmitter, receiver, and channel models adapted to the system are presented. Moreover, the details of the pre-equalizers designed for the system are described. In Section 3, the test results of the designed pre-equalizers implemented on the system are discussed. Finally, the article is concluded in Section 4.

## 2. VLC System Model

**Figure 1:**
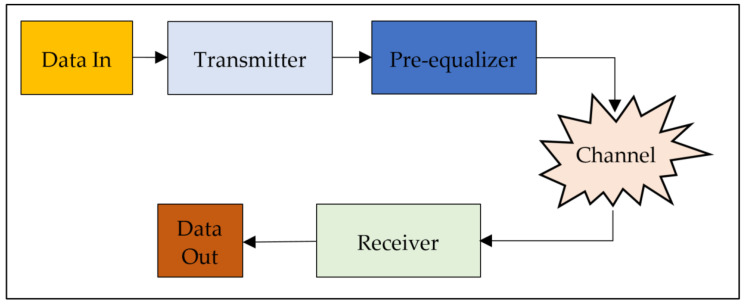
Structure of the VLC system modeled in MATLAB.

In order to design and implement the pre-equalizers, a VLC system was modeled in MATLAB. Figure 1 shows a block diagram of the model. As shown in the figure, the model basically consists of four main components, namely, a transmitter, a pre-equalizer, a channel, and a receiver. In the following, each of the components is described.

### 2.1. Transmitter and Receiver Modelling

In the system, it was aimed to achieve a data transmission rate of multiple Mbit/s. To this end, the Low Bandwidth OFDM Physical Layer (LB-PHY) algorithm defined in IEEE 802.15.13 was implemented. In the algorithm, OFDM-based modulation schemes were used. In general, since optical OFDM (O-OFDM) requires real-valued nonnegative symbols, it is not possible to use conventional OFDM directly in a VLC system [24]. Therefore, the DC-biased Optical OFDM (DCO-OFDM), which is the default modulation scheme of the IEEE 802.13.15-based LB-PHY algorithm, was used in the modelling of the transmitter and receiver.

**Figure 2:**
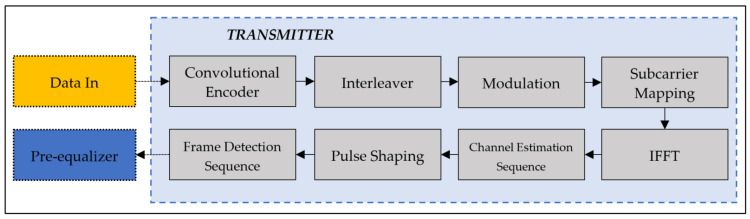
Structure of the transmitter model.

The structure of the transmitter model is shown in Figure 2. As shown in the figure, it is composed of eight stages, namely, Convolutional Encoder, Interleaver, Modulation, Subcarrier Mapping, Inverse Fast Fourier Transform (IFFT), Channel Estimation Sequence, Pulse Shaping, and Frame Detection Sequence.

In Convolutional Encoder stage, the data source is encoded with convolutional coding as forward error correction. Encoded bits are sourced to the Interleaver stage to spread the errors. Then, in the Modulation stage, the data stream is modulated with QAM mapping. In the Subcarrier Mapping stage, the QAM modulation symbols are mapped into the subcarriers with respect to Hermitian symmetry, and then they are passed through the Inverse Fast Fourier Transform (IFFT). Here, Hermitian symmetry is applied to the second half of the frame in order to ensure that the output of the IFFT is real. After the IFFT, a sequence of two identical OFDM training symbols is added to estimate the channel impulse response and provide additional fine-timing synchronization. In Pulse Shaping Filter stage, the data are upsampled by a factor of 8 and filtered before the ADC process. After the IFFT, the Frame Detection Sequence is added at the beginning of each packet to be used for packet detection and synchronization at the Receiver.

**Figure 3:**
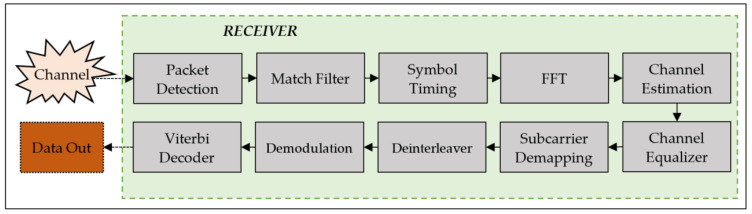
Structure of the receiver model.

On the other hand, the structure of the receiver model is shown in Figure 3. It is composed of 10 stages, namely, Packet Detection, Match Filter, Symbol Timing, FFT, Channel Estimation, Channel Equalizer, Subcarrier Demapping, Demodulation, Deinterleaver, and Viterbi Decoder.

In the receiver model, Packet Detection is responsible for finding the start point of the packet by using the original preamble sequence. Block works continuously until packet detection is succeeded. Once a packet is detected, the data are transferred to a Match Filter, which is the same filter as the Transmitter Pulse Shaping filter. The Match Filter is used to detect and correct the transmitted signal in the presence of a noisy and distorted received signal. After the incoming stream is filtered, it is sent to the Symbol Timing stage. 

The Symbol Timing stage has two functions: (a) Down sampling and (b) Frame detection. Firstly, the data is down-sampled by a factor of 8 through the maximum output energy method. With this method, the sampling points of each symbol are estimated. Frame Detection process is performed to down-sampled signals. The synchronization of OFDM symbols within a packet is also provided. The Frame Detection is performed by evaluating the cross correlation of CETS. The correlation result indicates the start and end points of an OFDM symbol. The cross-correlation operation is implemented as a FIR filter with 160 taps (coefficients), where the input of the filter is a part (400) of the detected (received) signal. Hence, after the Symbol Timing stage, synchronization within a packet is completed.

In the FFT stage, OFDM demodulation and cyclic prefix removal is applied. In order to recover the transmitted bits from the channel effects, the channel is first estimated in the Channel Estimation stage and then compensated in the Channel Equalizer. Here, the transmitted signal can be recovered by estimating the channel response at each subcarrier. Thus, the channel response of each subcarrier is estimated using the Channel Estimation Sequence, and this estimated response is then fed to the Channel Equalizer. Furthermore, a division/multiplication procedure is used to equalize the OFDM-demodulated signals. The estimated channel frequency response (inverse response of the channel) is multiplied by frequency domain OFDM symbols.

In the Subcarrier Demapping stage, the data subcarriers are demapped to data symbols, which are then sent to the Demodulation stage. In the Demodulation stage, the complex data symbols are demodulated to correct the disturbance effects of the channel. Next, the demodulated symbols are sent to the Deinterleaver stage. This is followed by a Viterbi Decoder, where the bit streams are decoded using a polynomial generator to create a Trellis Structure.

### 2.2. Channel Modelling

**Figure 4:**
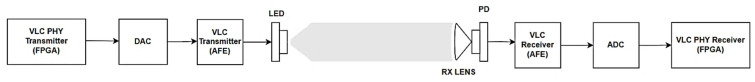
Illustration of fully operational VLC system.

In order to model the channel, firstly, real signal recordings were collected from an IEEE 802.15.13-compliant VLC system. As shown in Figure 4, the system consists of a PHY transmitter, a Digital-to-Analog Converter (DAC), a transmitter, a LED, channel, a photodetector (PD), a receiver, an Analog-to-Digital Converter (ADC), and a PHY receiver.

In the transmitting part of the system, a PHY transmitter was implemented on an FPGA device, where a bit stream was processed, which was then sourced to the DAC. The VLC transmitter (Analog Front-End: AFE) was used to drive the LED with the maximum possible amplitude in its linear region. Hence, the signal from the DAC was amplified, which was followed by the application of Bias Tee. It is important to note that the data turned out to be real due to Hermitian symmetry. However, since they are still bipolar, they are inappropriate for LED modulation. Hence, a DC bias is added to the data in order to shift the negative values to be positive and also to use the LED in its linear region before modulating the LED intensity. After the DC bias is added, the signal is transmitted by visible light with the help of the LED. Next, the signal was converted to visible light by the LED and transmitted through the LOS channel, where the LED and PD were fixed in height and distance. A reading light type LED was used in the test environment, and the bandwidth is approximately 4 MHz. Thus, it is the most bandwidth-limiting element in the channel.

In the receiving part of the system, a PD was used to capture the signal coming from the channel and convert it to the current. In order to convert the current to the voltage, a Transimpedance Amplifier (TIA) was used in VLC Receiver (AFE). The output of the receiver was then connected to the ADC to be processed by the PHY receiver. Similar to the PHY transmitter, the PHY receiver was also implemented on an FPGA device. It is worth noting that the sampling frequency of the ADC and DAC was set at 200 MHz. Since the up-sampling rate of the algorithm used in the transmitter model was set to 8, a 25 MHz bandwidth was chosen to be used in the system.

Channel response can be examined in three parts as Transmitter, Receiver, and Air. In this case, the Transmitter Response, $H_{T}\left(f\right)$, consists of DAC, LED, and VLC Transmitter responses:

(1) $$H_{T}\left(f\right)=H_{DAC}\left(f\right)H_{LED}\left(f\right)H_{TX}\left(f\right)$$

Receiver Response, $H_{R}\left(f\right),$ consists of ADC, PD, and VLC Receiver responses:

(2) $$H_{R}\left(f\right)=H_{ADC}\left(f\right)H_{PD}\left(f\right)H_{RX}\left(f\right)$$

when the effect of the air, $H_{A}\left(f\right)$, is added to the Transmitter and Receiver responses, the total channel response, H(f), can be expressed as follows:

(3) $$H\left(f\right)=H_{T}\left(f\right)H_{A}\left(f\right)H_{R}\left(f\right)$$

The signals obtained from the ADC process were recorded in order to observe the channel response so that the effects of the components, such as the DAC, LED, VLC transmitter, channel, PD, and VLC receiver, on the transmitted signal could be taken into account.

After collecting the recordings, they were post-processed in MATLAB, and the channel response of the system was obtained with help of CETS. More specifically, CETS consisted of two OFDM symbols where the disruptive effects of the channel could be observed. It should be noted that there are 64 subcarriers in the algorithm, since the subcarriers are Hermitian Symmetric. Therefore, only the gain of the first 32 subcarriers was used to create the channel model. The gain of each subcarrier was calculated by averaging the gain of two OFDM symbols. The calculated gains were then used to model the channel response, which was modelled as a 401 tap FIR filter. Finally, the channel model was integrated into the modelled VLC system.

### 2.3. Pre-Equalizer Designs

Two different pre-equalizers were designed to be used in the VLC system model. In the following, the designs of the proposed pre-equalizers are briefly introduced. The general purpose in Pre-Equalizer designs, assuming a frequency response of $H_{P}\left(f\right)$, is to eliminate the disruptive effects of the channel by applying the inverse of the channel response, assuming H(f). Once the channel response is estimated, then the frequency response of the pre-equalizer can be approximated:

(4) $$H_{P}\left(f\right)≅1/H\left(f\right)$$

Then, the overall system response can be written as:

(5) $$S_{R}\left(f\right)=S\left(f\right)H\left(f\right)H_{P}\left(f\right)$$

where; S(f) is the desired response, while $S_{R}\left(f\right)$ is the received response after the pre-equalization. Then, the design of the pre-equalizer is needed to remove channel-induced effects. A perfectly estimated channel response along with a perfectly constructed pre-equalizer remove all channel effects perfectly. In practice, however, it is almost impossible, especially in multi-carrier systems. Here, we implement two FIR-based pre-equalizers and demonstrate that channel effects are greatly reduced in a fully operational VLC system, a multi-carrier operated system.

#### 2.3.1. Pre-Equalizer 1

For the first pre-equalizer, the main idea was to use the inverse of the gain responses of each subcarrier that could be obtained from the CETS. Therefore, the same method used to collect signal recordings for channel modelling was utilized in the first design. In this context, after the modelled channel was integrated between the transmitter and receiver in the VLC system model, the transfer was initiated by the transmitter, and CETS was recorded in the receiver. The channel response of each subcarrier was then obtained from CETS, and the inverse of these responses was used to create a 201 tap FIR filter as a pre-equalizer.

**Figure 5:**
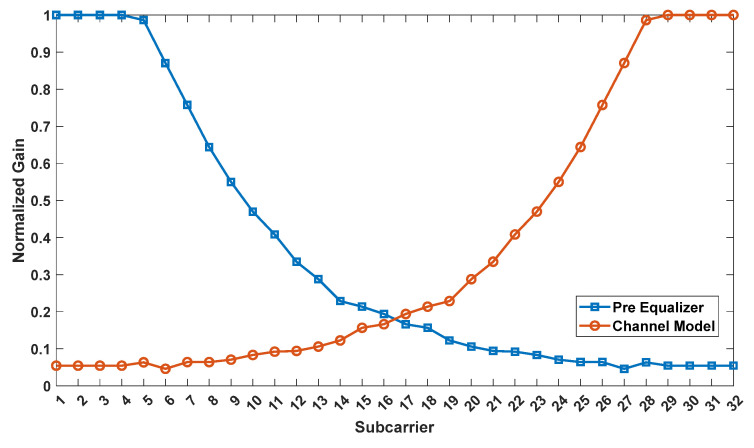
Subcarrier gains of the channel and the first pre-equalizer.

The comparison of the normalized gain responses of the first 32 subcarriers in the channel and the first pre-equalizer are shown in Figure 5. It can be clearly observed from the figure that the channel model behaves similar to a low-pass filter, whereas the pre-equalizer behaves similar to a high-pass filter.

#### 2.3.2. Pre-Equalizer 2

In the design of the second pre-equalizer, single tone sinusoidal signals were generated with a frequency range from 1 MHz to 25 MHz in 1 MHz steps. The generated signals were then transmitted to the channel in the VLC system model. A FFT process was applied to the signals received from the channel. Thus, the amplitude value corresponding to the relevant frequency was obtained. In this case, the channel response was also obtained for each frequency in the bandwidth. Hence, the 201 tap pre-equalizer filter was created by using the inverse of the calculated channel response.

## 3. Simulations and Results

The proposed pre-equalizers were tested on the VLC system designed in the previous section to evaluate their feasibility in terms of BER performance. For this purpose, simulations were conducted in MATLAB. In the simulations, a random bit stream, consisting of 1.6376 × 10^3^ bits, was generated and transmitted to the designed pre-equalizer filters. Then, the subcarriers, which were equalized with respect to the frequency response, were amplified 16 times and sent to the channel. The data formed after the channel were passed through the receiver. Finally, a BER analysis was carried out. During the BER analysis, the maximum packet size defined in the IEEE 802.13.15 (2047 bytes) standard was used. The simulations were carried out 1000 times for the VLC system with and without pre-equalizer designs.

On the other hand, the LB-PHY algorithm defined in the IEEE 802.15.13 standard supports BPSK, QPSK, 16-QAM, and 64-QAM modulation schemes. Among these schemes, due to its higher bandwidth efficiency rate compared to other lower order QAMs, 64-QAM was chosen to be used in the simulations. In addition, in order to verify the feasibility of the proposed pre-equalizers in other higher-order QAMs, 256-QAM was also used in the simulations, although it is not supported by the standard. The code rate was fixed at ¾ before conducting the simulations. 

**Figure 6:**
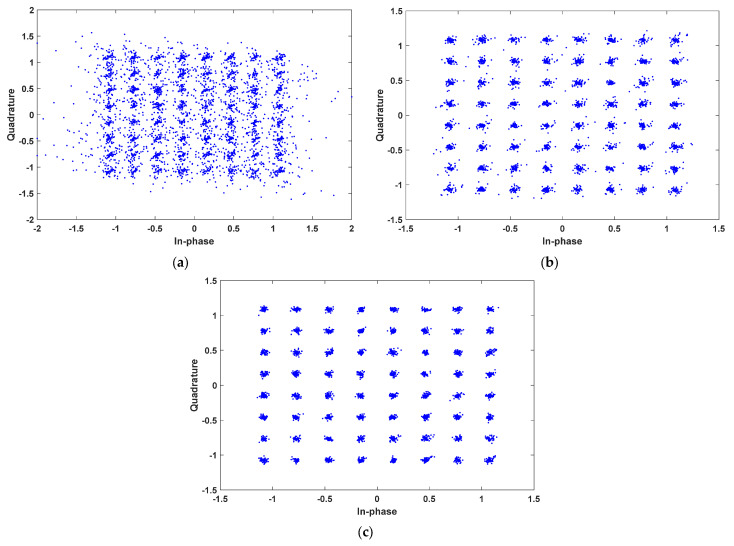
Constellation diagrams of 64-QAM for the VLC system: (**a**) without a pre-equalizer; (**b**) using the first pre-equalizer; and (**c**) using the second pre-equalizer.

**Figure 7:**
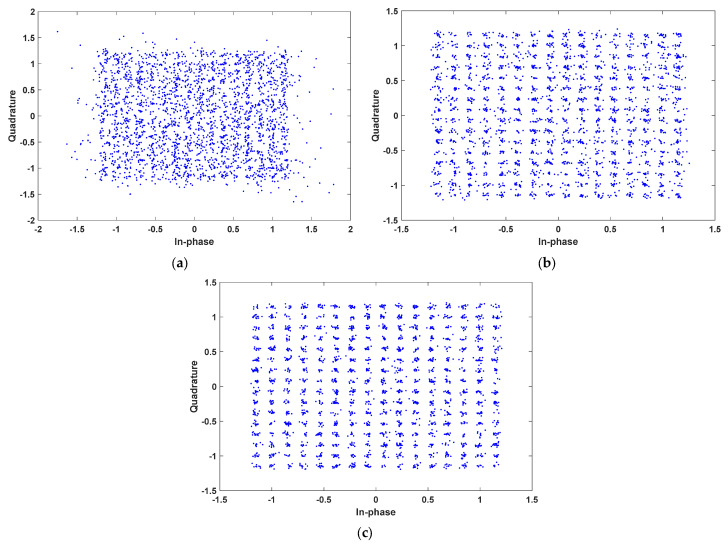
Constellation diagrams of 256-QAM for the VLC system: (**a**) without a pre-equalizer; (**b**) using the first pre-equalizer; and (**c**) using the second pre-equalizer.

The experimental results in terms of BER are listed in Table 1. Constellation diagrams of 64-QAM for the VLC system without pre-equalizer, with first pre-equalizer, and with second pre-equalizer are shown in Figure 6a–c, respectively. Moreover, constellation diagrams of 256-QAM for the VLC system without pre-equalizer, with first pre-equalizer, and with second pre-equalizer are shown in Figure 7a–c, respectively.

The results achieved from the simulations can be discussed in two aspects: (1) the effects of the proposed pre-equalizers on the system’s BER performance, (2) the BER performance of the system with the proposed equalizers under bandwidth-efficient modulation schemes, such as 64-QAM and 256-QAM. From the results listed in Table 1, it is clear that the BER performance of the system without a pre-equalizer is poor under a higher level of QAM modulation schemes. Specifically, the BER values of the system without pre-equalizers are 19,900 × 10^−5^ and 35,000 × 10^−5^ for 64-QAM and 256-QAM, respectively. Yet, the BER performance of the system is considerably improved with the proposed pre-equalizers.

As can be seen in Table 1, when 64-QAM is used, the BER value of the system with the first equalizer is found to be 0.438 × 10^−5^. However, when 256-QAM is used, the BER value of the system with the first equalizer is reduced, which is found to be 2700 × 10^−5^. Nevertheless, the achieved BER performance is acceptable, which allows the first equalizer to be used for 256-QAM signals.

On the other hand, for the system with the second equalizer, a BER of 0 is achieved under 64-QAM, while a BER of 6.65 × 10^−5^ is achieved under 256-QAM. This suggests that the system with the second equalizer still attains effective BER performance, although a higher level of QAM modulation scheme is used. Hence, it is evident that the system with the second pre-equalizer offers superior BER performance compared to the system with the first equalizer.

Simulation results show that both pre-equalizer designs significantly improve the BER performance of the VLC system. However, further discussions are needed to address the limitations in the design of the proposed pre-equalizers. In the next section, the proposed pre-equalizers are discussed in terms of design requirements.

On the other hand, again, note that the proposed pre-equalizers are fully based on implemented IEEE 802.15.13 standard, and, moreover, the VLC system implemented in this context is likely to be integrated within a platform. Based on our study survey, as a fully operational VLC model (using the relevant standard) employing pre-equalization is available for comparison, it is not easy to compare directly with available pre-equalizer performances. However, based on the constellation diagrams, as well as BER performance, we could conclude that the proposed pre-equalizer achieves quite satisfactory rates on a fully operational VLC system based on the standard.

## 4. Discussion

Although simulation results provide promising evidence for the designed equalizers, there might be some challenges and difficulties in their design phase. In this section, these concerns are briefly discussed to provide useful insights that may be ultimately important while making decisions in the design of the proposed pre-equalizers.

In the design phase of the first pre-equalizer, since the CETS is used to obtain the inverse of the gain responses of each subcarrier, it is necessary to apply a post-process approach. In this approach, the received signals (recordings) should be processed in a software environment, such as MATLAB, by employing packet detection and symbol timing algorithms to remove CETS from the recordings, and then, FFT needs to be applied. As an alternative to the post-process, an extension that calculates the filter coefficients could be added to the PHY algorithm on hardware, such as a FPGA. This results in a dynamic pre-equalizer that enables us to calculate the filter coefficients easily, even if the transmission channel is changed. However, its implementation might be difficult in practice. Therefore, using the post-process approach is an easier way that could be preferred for the design of the first pre-equalizer.

Since the design of the second pre-equalizer is based on the use of sinusoidal signals, it is not necessary to apply a post-process. For this reason, the second pre-equalizer can be realized with the recordings collected from a real setup. Hence, a testing environment (measurement setup) is required to be built. In this case, however, it is not possible to provide a pre-equalizer in a dynamic structure. Thus, the filter coefficients need to be recalculated when the transmission channel is changed.

Overall, it can be concluded that, although the use of the second pre-equalizer in the VLC system provides lower BERs, its design and implementation might be costly. At the expense of relatively higher BERs, the first design can be selected to be used in the VLC system. It should be noted that, in its design, the post-process approach could be used for the sake of simplicity.

## 5. Conclusions

In this study, two digital pre-equalizers were proposed to be used in VLC systems based on the IEEE 802.15.13 standard. The proposed pre-equalizers were implemented on a VLC system modeled in MATLAB. Simulations were conducted to evaluate the BER performance of the system. According to the results, it has been shown that the BER performance of the system could be improved with the proposed pre-equalizers. In fact, the system with the second pre-equalizer provides better BER performance compared to the system with the first equalizer. However, the use of the first equalizer offers a low-cost alternative for the VLC system.

## Figures and Tables

**Table 1:** Simulation results in terms of BER.

System	BER	
	64-QAM	256-QAM
Without pre-equalizer	1990 × 10^−5^	35,000 × 10^−5^
First pre-equalizer	0.438 × 10^−5^	2700 × 10^−5^
Second pre-equalizer	0	6.65 × 10^−5^

## Data Availability

The data presented in this study are available upon request from the corresponding author.

## References

[1] Haas H., Yin L., Wang Y., Chen C. (2015). What Is Lifi?. J. Light. Technol..

[2] Uysal M., Nouri H. Optical Wireless Communications—An Emerging Technology. Proceedings of the 2014 16th international conference on transparent optical networks (ICTON).

[3] Khan L.U. (2017). Visible Light Communication: Applications, Architecture, Standardization and Research Challenges. Digit. Commun. Netw..

[4] Rehman S.U., Ullah S., Chong P.H.J., Yongchareon S., Komosny D. (2019). Visible Light Communication: A System Perspective—Overview and Challenges. Sensors.

[5] Uysal M., Ghassemlooy Z., Bekkali A., Kadri A., Menouar H. (2015). Visible Light Communication for Vehicular Networking: Performance Study of a V2V System Using a Measured Headlamp Beam Pattern Model. IEEE Veh. Technol. Mag..

[6] Rapheal M., Vargheese V., Joy S., Xavi S. (2016). Visible Light Communication in Defence and Security. Int. Res. J. Eng. Technol. (IRJET).

[7] Ali M.F., Jayakody D.N.K., Li Y. (2022). Recent Trends in Underwater Visible Light Communication (UVLC) Systems. IEEE Access.

[8] Oyewobi S.S., Djouani K., Kurien A.M. (2022). Visible Light Communications for Internet of Things: Prospects and Approaches, Challenges, Solutions and Future Directions. Technologies.

[9] Yu T.-C., Huang W.-T., Lee W.-B., Chow C.-W., Chang S.-W., Kuo H.-C. (2021). Visible Light Communication System Technology Review: Devices, Architectures, and Applications. Crystals.

[10] O’brien D.C., Zeng L., Le-Minh H., Faulkner G., Walewski J.W., Randel S. Visible Light Communications: Challenges and Possibilities. Proceedings of the 2008 IEEE 19th International Symposium on Personal, Indoor and Mobile Radio Communications.

[11] Liu Y.F., Chang Y.C., Chow C.-W., Yeh C.H. (2011). Equalization and Pre-Distorted Schemes for Increasing Data Rate in in-Door Visible Light Communication System. Proceedings of the 2011 Optical Fiber Communication Conference and Exposition and the National Fiber Optic Engineers Conference, Los Angeles, CA, USA, 6–10 March 2011.

[12] Zhang M., Zhang Z. Fractionally Spaced Equalization in Visible Light Communication. Proceedings of the 2013 IEEE Wireless Communications and Networking Conference (WCNC).

[13] Liu Y.-F., Yeh C.-H., Chow C.-W., Huang P.-Y., Liu Y. (2013). Demonstration of Using Digital FIR Filter and Matched Filter to Increase Data Rate in Visible Light Communication. Proceedings of the Broadband Access Communication Technologies VII, San Francisco, CA, USA, 5–7 February 2013.

[14] Haigh P.A., Ghassemlooy Z., Rajbhandari S., Leitgeb E. A 100 Mb/s Visible Light Communications System Using a Linear Adaptive Equalizer. Proceedings of the 2014 19th European Conference on Networks and Optical Communications-(NOC).

[15] Zhou Y., Liang S., Chen S., Huang X., Chi N. 2.08 Gbit/s Visible Light Communication Utilizing Power Exponential Pre-Equalization. Proceedings of the 2016 25th Wireless and Optical Communication Conference (WOCC).

[16] Han S., Wang C., Li G., Chi N. A 427.5 Mbps Automotive Headlight Visible Light Communication System Utilizing 64QAM-DMT Modulation with Software Pre-Equalization. Proceedings of the 2019 IEEE/CIC International Conference on Communications in China (ICCC).

[17] Mathias L.C., Marinello Filho J.C., Abrao T. (2019). Predistortion and Pre-Equalization for Nonlinearities and Low-Pass Effect Mitigation in OFDM-VLC Systems. Appl. Opt..

[18] Surampudi A., Collins S. Simple Digital Pre-Equalization of VLC Links. Proceedings of the 2020 IEEE Photonics Conference (IPC).

[19] Jin J., Wang J., Chen D., Lu H. (2020). Experimental Implementation of Digital Equalizer for Multilevel Signal in Visible Light Communication. Opt. Eng..

[20] Chen C., Nie Y., Liu M., Du Y., Liu R., Wei Z., Fu H.Y., Zhu B. (2021). Digital Pre-Equalization for OFDM-Based VLC Systems: Centralized or Distributed?. IEEE Photonics Technol. Lett..

[21] Kisacik R., Yagan M.Y., Uysal M., Pusane A.E., Yalcinkaya A.D. (2021). A New LED Response Model and Its Application to Pre-Equalization in VLC Systems. IEEE Photonics Technol. Lett..

[22] Yang C., Han D., Zhang M., Wang L., Jia P., Jiang X., Huang X. (2023). Visible Light Communication-Channel-Adaptive Digital Pre-Equalization Scheme Based on a Deep Learning Model. Opt. Eng..

[23] Ramadhan M.A., Tanudjaja G.H., Setiawan E., Adiono T., Sutisna N., Mulyawan R., Syafalni I. Design and Implementation of a Pre-Equalizer for Visible Light Communication. Proceedings of the 2021 International Symposium on Intelligent Signal Processing and Communication Systems (ISPACS).

[24] Le Minh H., O’Brien D., Faulkner G., Zeng L., Lee K., Jung D., Oh Y. (2008). High-Speed Visible Light Communications Using Multiple-Resonant Equalization. IEEE Photonics Technol. Lett..

[25] Le Minh H., O’Brien D., Faulkner G., Zeng L., Lee K., Jung D., Oh Y., Won E.T. (2009). 100-Mb/s NRZ Visible Light Communications Using a Postequalized White LED. IEEE Photonics Technol. Lett..

[26] Kısacık R., Yagan M.Y., Uysal M., Pusane A.E., Baykas T., Dundar G., Yalcinkaya A.D. (2022). A 130 Nm CMOS Receiver for Visible Light Communication. J. Light. Technol..

[27] Yeh C.H., Chow C.W., Liu Y.F., Huang P.Y. (2013). Simple Digital FIR Equalizer Design for Improving the Phosphor LED Modulation Bandwidth in Visible Light Communication. Opt. Quantum Electron..

[28] (2023). IEEE Approved Draft Standard for Multi-Gigabit per Second Optical Wireless Communications (OWC), with Ranges up to 200 Meters, for Both Stationary and Mobile Devices.

